# Forecasting Influenza with the Long Short-Term Memory Model: Results from the 2023-2024 Influenza Season

**Published:** 2025-04-20

**Authors:** Sneha P. Cherukuri, Mark L. Bova, Shaylee P. Mehta, Christian T. Bautista

**Affiliations:** Armed Forces Health Surveillance Division, Integrated Biosurveillance Branch, Silver Spring, MD: Ms. Cherukuri, Mr. Bova, Ms. Mehta, Dr. Bautista

Timely detection of infectious diseases and health threats is of increasing importance, particularly for U.S. military service members. Existing surveillance systems are hindered, however, by a 1-to 2-week delay between actual disease outbreaks and release of surveillance data. ^1^ To address this challenge, since 2019 the Integrated Biosurveillance (IB) Branch of the Armed Forces Health Surveillance Division has conducted forecasting activities during influenza season to provide early warning and increased awareness of potential health risks to the Department of Defense (DOD) enterprise. ^2^ At the end of each influenza season, IB evaluates the performance of the individual forecasting models and assesses potential integration of new algorithms to improve forecasting capabilities for the next influenza season.

The Long Short-Term Memory (LSTM) model is a machine-learning method with potential to improve forecasting accuracy for respiratory disease surveillance. ^3^ The LSTM model is a recurrent neural network model that can be used in almost all modeling fields. LSTM has the capacity to selectively add new information and forget previously accumulated information. While LSTM models are well-established, their performance in forecasting influenza encounters utilizing DOD surveillance data has not been studied. This report assesses the performance of the LSTM model for possible inclusion in future DOD influenza forecasting analyses.

## Methods

Influenza encounters were defined as outpatient visits with an International Classification of Diseases, 10th Revision (ICD-10) discharge diagnosis code, with codes J09 through J11 selected and identified for influenza encounters. Outpatient influenza encounter data from Military Health System (MHS) beneficiaries were collected weekly during the 2023-2024 influenza season from all U.S. military hospitals and clinics. Total outpatient encounter data were obtained from the DOD's Electronic Surveillance System for the Early Notification of Community-based Epidemics (ESSENCE). The percentage of outpatient influenza encounters was calculated as the weekly percentage of total outpatient encounters.

Short-term, 1-2-week forecasts were previously generated by the IB Branch each week during the influenza season for the U.S., including all military hospitals and clinics for 2023 epidemiological week (EW) 40 through 2024 EW 20. Forecasts were generated weekly using various time series and machine learning models, including autoregressive integrated moving average (ARIMA), error-trend-seasonality (ETS), exponentially weighted moving average (EWMA), naïve (NAÏVE), neural network (NNET), poisson (POISSON), prophet (PROPHET), random forest (RF), time series linear model (TSLM), and vector autoregressive (VAR) model. An ensemble model (ENSEMBLE) was created as an average of all the forecasting models used.

Short-term, 1-2-week LSTM model forecasts were generated for percentages of MHS influenza encounters for each week of the 2023-2024 influenza season by utilizing training data from the previous influenza season (2022 EW 40 through 2023 EW 20). Forecast horizons, the timeframe for which a forecast is made, were defined for 1 week, 2 weeks, and 1-2 weeks ahead. To validate the model, the data were separated into training and testing sets for each EW of evaluation. Training loss was calculated using mean squared error (MSE). Key hyper-parameters including number of hidden units (50), dropout rate (0.2), and an adaptive retrospective period were used to improve model performance.

Weekly forecasts were then compared with observed values from each EW using the weighted interval score (WIS) ^4^ and absolute percentage error (APE). Scores from the LSTM model were then combined with all previously generated model scores to assess model performance.

All analyses and data processing used R version 4.4.2. LSTM models were created using the “torch” package in R, an open-source machine learning framework based on PyTorch. ^5^

## Results

WIS, log-transformed WIS, and APE were calculated for 1,924 total forecasts. The average training loss per evaluation week for the LSTM model was 0.5. Median log-transformed WIS and median APE are shown in the **Table** for each model as well as 1-week, 2-week, and combined 1-2-week forecasts. The LSTM model had the lowest median log-transformed WIS for all forecasting horizons: 1 week (0.3), 2 weeks (0.4), and combined 1-2 weeks (0.4). The VAR model had the lowest median APE for all forecasting horizons (37.5%). **Figure 1a** presents forecasts with 95% confidence interval (CI) bands for the LSTM and ENSEMBLE models over the study period. During 2023 EWs 51 and 52, observed influenza encounter percentages peaked at 0.5% and 0.8%, respectively. The LSTM and ENSEMBLE models under-predicted values, however, with estimates ranging from 0.17% to 0.2% during this period. **Figure 1b** displays a grouped boxplot of log WIS for each forecast target for all models, ranked by median log WIS. The LSTM model had the lowest log WIS, while the POISSON model had the highest.

**TABLE.** Median Weighted Interval Score (WIS) and Median Absolute Percent Error for Outpatient Influenza Encounter Forecasts in the Military Health System Population

	1 Week Ahead		2 Weeks Ahead		1-2 Weeks Ahead	
Model	Median Log (WIS)	Median Absolute Percent Error (%)	Median Log (WIS)	Median Absolute Percent Error (%)	Median Log (WIS)	Median Absolute Percent Error (%)
LSTM	0.3	45.9	0.4	43.7	0.4	45.2
EWMA	0.4	37.5	0.5	42.9	0.4	37.5
VAR	0.4	37.5	0.5	37.5	0.4	37.5
NAIVE	0.4	37.5	0.8	42.9	0.5	37.5
ETS	0.4	37.5	0.8	42.9	0.6	37.5
NNET	0.4	41.2	0.7	45.2	0.6	42.9
ARIMA	0.6	42.9	0.8	42.9	0.7	42.9
PROPHET	0.7	42.9	0.8	38.5	0.7	39.4
TSLM	2.4	65.5	2.4	65.5	2.4	65.5
POISSON	10.3	64.9	10.7	64.9	10.5	64.3
RF	NA*	39.2	NA	46.5	NA	42.9

Abbreviations: NA, not available; MHS, Military Health System.

**FIGURE 1a.**
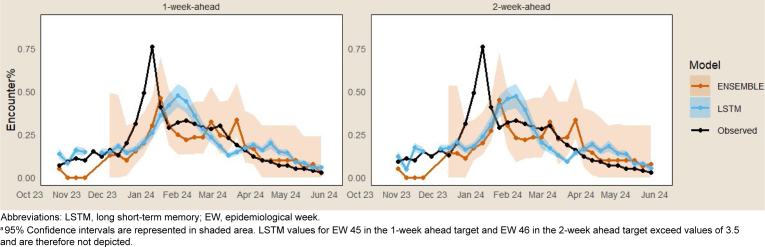
Influenza Encounter Percentage by Forecast Target, Military Health System, November 2023–June 2024

**FIGURE 1b.**
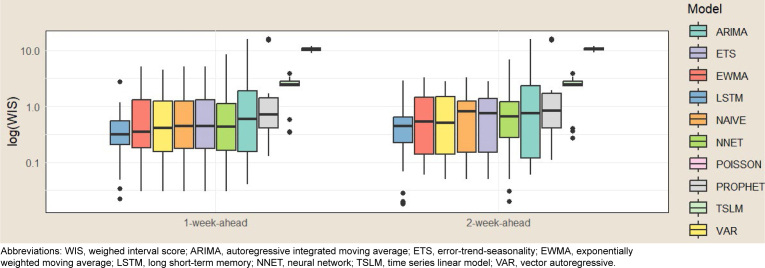
Weighted Interval Score by Forecast Target

## Discussion

Our analyses indicate that LSTM had the lowest log WIS among the individual models for all forecasting horizons, resulting in more accurate forecasts. These findings align with previous studies that successfully used LSTM models to fore-cast influenza-like illness and influenza hospitalizations. ^6 , 7^ Neither the LSTM nor ENSEMBLE models accurately predicted the peak period, 2023 EWs 51-52 (December 17-30), however. This could be due to the utilization of 2022-2023 influenza season data for the training data, as recent seasonal influenza patterns have exhibited significantly higher peaks earlier in the season compared to influenza seasons prior to the COVID-19 pandemic. ^8 , 9^ To improve influenza peak period forecasts, training data may need to include multiple years, before and after the COVID-19 pandemic, as part of further analysis.

This study had some limitations. First, this study did not employ a formal cross-validation method to optimize hyper-parameters and construct the best-performing LSTM model, which may have contributed to poor predictions, particularly in the early weeks of the study period. Further research is needed to optimize the LSTM model for influenza encounter predictions. Second, some WIS values were found to be 0, indicating that the estimated value was an exact match to the observed value. Scores equal to 0 should be interpreted with caution, as those values may be due to overconfidence and result in an undefined log-transformed WIS. ^10^ Consequently, WIS values equal to 0 were excluded from the calculation of log-transformed WIS, but this may have introduced bias by excluding forecasts that were very close to actual values. Third, it is not possible to state with confidence that these results are generalizable to other respiratory diseases or related metrics such as hospitalizations, admission rates, or case rates. Lastly, this analysis does not reflect changes after the 2023-2024 influenza season to improve forecasting, such as the removal of the ETS, EWMA, PROPHET, and TSLM models. Although the LSTM model outperformed several models included in the ENSEMBLE model, it is likely the ENSEMBLE model will perform better for the 2024-2025 influenza season.

The findings of this study demonstrate that the addition of the LSTM model improves the short-term forecasting performance of the ENSEMBLE model for outpatient influenza encounter data, which is commonly used to assess the activity intensity of this respiratory disease within the MHS population. Further research is recommended to determine the performance of the LSTM model for other respiratory infections, including COVID-19.
